# 4-Benzyl-8-phenyl-1-thia-4-aza­spiro­[4.5]decan-3-one

**DOI:** 10.1107/S1600536812015358

**Published:** 2012-04-18

**Authors:** Hoong-Kun Fun, Tze Shyang Chia, Poovan Shanmugavelan, Alagusundaram Ponnuswamy

**Affiliations:** a X-ray Crystallography Unit, School of Physics, Universiti Sains Malaysia, 11800 USM, Penang, Malaysia; b Department of Organic Chemistry, School of Chemistry, Madurai Kamaraj University, Madurai 625 021, Tamil Nadu, India

## Abstract

In the title compound, C_21_H_23_NOS, the thia­zolidine ring adopts a twist conformation about one of its C—S bonds, while the cyclo­hexane ring has a chair conformation. The S and N atoms attached to the spiro C atom are in axial and equatorial orientations, respectively. The thia­zolidine ring forms dihedral angles of 86.24 (14) and 31.82 (15)° with the directly attached and remote terminal benzene rings, respectively. The dihedral angle between the two terminal benzene rings is 86.74 (14)°. In the crystal, the only significant directional inter­action is a weak C—H⋯π bond, which generates [010] chains.

## Related literature

For the pharmacological activity of spiro­thia­zolidin-4-ones, see: Singh *et al.* (2006 ▶); Kasimogullari & Cesur (2004 ▶); Dandia *et al.* (2004 ▶); Sahu *et al.* (2006 ▶). For a related structure, see: Akkurt *et al.* (2008 ▶). For ring puckering parameters, see: Cremer & Pople (1975 ▶). For the stability of the temperature controller used for data collection, see: Cosier & Glazer (1986 ▶). For standard bond lengths, see: Allen *et al.* (1987 ▶).
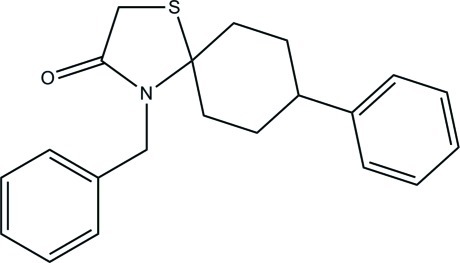


## Experimental

### 

#### Crystal data

C_21_H_23_NOS
*M* *_r_* = 337.46
Monoclinic, 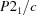

*a* = 9.8299 (9) Å
*b* = 15.3823 (14) Å
*c* = 12.0833 (10) Å
β = 108.717 (4)°
*V* = 1730.4 (3) Å^3^
*Z* = 4
Mo *K*α radiation
μ = 0.19 mm^−1^
*T* = 100 K
0.33 × 0.21 × 0.14 mm

#### Data collection

Bruker SMART APEXII CCD diffractometer
Absorption correction: multi-scan (*SADABS*; Bruker, 2009 ▶) *T*_min_ = 0.939, *T*_max_ = 0.973
10508 measured reflections
3534 independent reflections
2144 reflections with *I* > 2σ(*I*)
*R*_int_ = 0.080

#### Refinement

*R*[*F* ^2^ > 2σ(*F* ^2^)] = 0.062
*wR*(*F* ^2^) = 0.145
*S* = 1.01
3534 reflections
217 parameters
H-atom parameters constrained
Δρ_max_ = 0.71 e Å^−3^
Δρ_min_ = −0.46 e Å^−3^

### 

Data collection: *APEX2* (Bruker, 2009 ▶); cell refinement: *SAINT* (Bruker, 2009 ▶); data reduction: *SAINT*; program(s) used to solve structure: *SHELXTL* (Sheldrick, 2008 ▶); program(s) used to refine structure: *SHELXTL*; molecular graphics: *SHELXTL*; software used to prepare material for publication: *SHELXTL* and *PLATON* (Spek, 2009 ▶).

## Supplementary Material

Crystal structure: contains datablock(s) global, I. DOI: 10.1107/S1600536812015358/hb6732sup1.cif

Structure factors: contains datablock(s) I. DOI: 10.1107/S1600536812015358/hb6732Isup2.hkl

Supplementary material file. DOI: 10.1107/S1600536812015358/hb6732Isup3.cml

Additional supplementary materials:  crystallographic information; 3D view; checkCIF report

## Figures and Tables

**Table 1:** Hydrogen-bond geometry (Å, °) *Cg*1 is the centroid of the C1–C6 ring.

*D*—H⋯*A*	*D*—H	H⋯*A*	*D*⋯*A*	*D*—H⋯*A*
C17—H17*A*⋯*Cg*1^i^	0.95	2.72	3.565 (3)	149

Symmetry code: (i) 
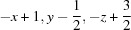
.

**Fig. 1.**
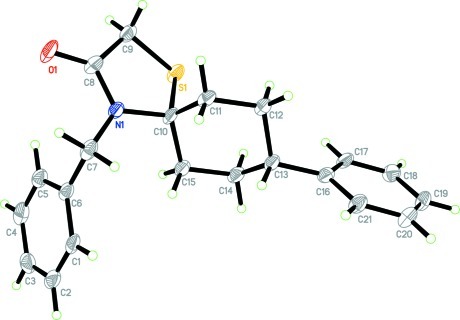
The molecular structure of the title compound with 50% probability displacement ellipsoids.

## supplementary crystallographic information

### Comment

Spirothiazolidin-4-ones are well known to possess varied pharmacological activities such as anti-fungal (Singh *et al.*, 2006), anti-mycobacterial (Kasimogullari & Cesur, 2004), anti-TB (Dandia *et al.*, 2004), and anti-bacterial (Sahu *et al.*, 2006) properties. As a part of our studies in this area, we now describe the synthesis and structure of the title compound, (I).

The asymmetric unit of (I) is shown in Fig. 1. The central thiazolidine ring (S1/N1/C8–C10) is twisted with deviations from the least-squares plane of 0.199 (1) and -0.211 (3) Å for atoms S1 and C10, respectively. The cyclohexane ring (C10–C15) adopts a chair conformation with puckering parameters (Cremer & Pople, 1975), *Q* = 0.583 (3) Å, θ = 173.4 (3)° and φ = 347 (2)°. The thiazolidine ring forms dihedral angles of 86.24 (14) and 31.82 (15)° with the terminal C1–C6 and C16–C21 benzene rings, respectively. The dihedral angle between the two terminal benzene rings is 86.74 (14)°. Bond lengths and angles are comparable to a related structure (Akkurt *et al.*, 2008).

The only significant intermolecular interaction in the crystal is a weak C—H···*Cg*1 interaction (Table 1) where *Cg*1 is the centroid of C1–C6 ring.

### Experimental

A mixture of triphenyl phosphine (0.43 g, 1.1 mmol), benzyl azide (0.20 g, 1.0 mmol), 4-phenylcylohexanone (0.28 g, 1.0 mmol) and mercaptoacetic acid (0.15 g, 1.1 mmol) was heated to reflux in acetonitrile (5 ml) for 4 h. The reaction mixture was allowed to stand at room temperature. After the solvent has been evaporated, the residue was purified by column chromatography using silica gel as the stationary phase (60–120 mesh) and petroleum ether-ethyl acetate (93:7) as the mobile phase to afford the title compound. Yield: 0.48 g (95%); *M*.p.: 130–131 °C. Colourless crystals were obtained by recrystallization from ethanol solution.

### Refinement

All H atoms were positioned geometrically [C—H = 0.95, 0.99 or 1.00 Å] and refined using a riding model with *U*_iso_(H) = 1.2 *U*_eq_(C).

### Crystal data

**Table:**

C_21_H_23_NOS	*F*(000) = 720
*M**_r_* = 337.46	*D*_x_ = 1.295 Mg m^−^^3^
Monoclinic, *P*2_1_/*c*	Mo *K*α radiation, λ = 0.71073 Å
Hall symbol: -P 2ybc	Cell parameters from 2184 reflections
*a* = 9.8299 (9) Å	θ = 2.6–29.6°
*b* = 15.3823 (14) Å	µ = 0.19 mm^−^^1^
*c* = 12.0833 (10) Å	*T* = 100 K
β = 108.717 (4)°	Block, colourless
*V* = 1730.4 (3) Å^3^	0.33 × 0.21 × 0.14 mm
*Z* = 4	

### Data collection

**Table:**

Bruker SMART APEXII CCD diffractometer	3534 independent reflections
Radiation source: fine-focus sealed tube	2144 reflections with *I* > 2σ(*I*)
Graphite monochromator	*R*_int_ = 0.080
φ and ω scans	θ_max_ = 26.5°, θ_min_ = 2.2°
Absorption correction: multi-scan (*SADABS*; Bruker, 2009)	*h* = −12→11
*T*_min_ = 0.939, *T*_max_ = 0.973	*k* = −16→19
10508 measured reflections	*l* = −15→15

### Refinement

**Table:**

Refinement on *F*^2^	Primary atom site location: structure-invariant direct methods
Least-squares matrix: full	Secondary atom site location: difference Fourier map
*R*[*F*^2^ > 2σ(*F*^2^)] = 0.062	Hydrogen site location: inferred from neighbouring sites
*wR*(*F*^2^) = 0.145	H-atom parameters constrained
*S* = 1.01	*w* = 1/[σ^2^(*F*_o_^2^) + (0.0622*P*)^2^ + 0.185*P*] where *P* = (*F*_o_^2^ + 2*F*_c_^2^)/3
3534 reflections	(Δ/σ)_max_ < 0.001
217 parameters	Δρ_max_ = 0.71 e Å^−^^3^
0 restraints	Δρ_min_ = −0.46 e Å^−^^3^

### Special details

**Table:**

Experimental. The crystal was placed in the cold stream of an Oxford Cryosystems Cobra open-flow nitrogen cryostat (Cosier & Glazer, 1986) operating at 100.0 (1) K.
Geometry. All e.s.d.'s (except the e.s.d. in the dihedral angle between two l.s. planes) are estimated using the full covariance matrix. The cell e.s.d.'s are taken into account individually in the estimation of e.s.d.'s in distances, angles and torsion angles; correlations between e.s.d.'s in cell parameters are only used when they are defined by crystal symmetry. An approximate (isotropic) treatment of cell e.s.d.'s is used for estimating e.s.d.'s involving l.s. planes.
Refinement. Refinement of *F*^2^ against ALL reflections. The weighted *R*-factor *wR* and goodness of fit *S* are based on *F*^2^, conventional *R*-factors *R* are based on *F*, with *F* set to zero for negative *F*^2^. The threshold expression of *F*^2^ > σ(*F*^2^) is used only for calculating *R*-factors(gt) *etc*. and is not relevant to the choice of reflections for refinement. *R*-factors based on *F*^2^ are statistically about twice as large as those based on *F*, and *R*- factors based on ALL data will be even larger.

### Fractional atomic coordinates and isotropic or equivalent isotropic displacement parameters (Å^2^)

**Table:**

	*x*	*y*	*z*	*U*_iso_*/*U*_eq_	
S1	0.33380 (9)	−0.00046 (5)	0.85069 (6)	0.0253 (2)	
O1	0.3626 (2)	0.18832 (13)	1.06649 (16)	0.0320 (6)	
N1	0.3843 (3)	0.16539 (15)	0.88513 (19)	0.0211 (6)	
C1	0.6646 (3)	0.29768 (18)	0.8312 (2)	0.0241 (7)	
H1A	0.6047	0.3262	0.7633	0.029*	
C2	0.8123 (4)	0.3003 (2)	0.8566 (3)	0.0296 (8)	
H2A	0.8531	0.3314	0.8071	0.035*	
C3	0.9005 (4)	0.2575 (2)	0.9542 (3)	0.0309 (8)	
H3A	1.0017	0.2574	0.9702	0.037*	
C4	0.8401 (4)	0.2147 (2)	1.0284 (3)	0.0310 (8)	
H4A	0.9001	0.1868	1.0968	0.037*	
C5	0.6923 (3)	0.21282 (19)	1.0028 (2)	0.0257 (7)	
H5A	0.6517	0.1830	1.0536	0.031*	
C6	0.6026 (3)	0.25385 (18)	0.9040 (2)	0.0211 (7)	
C7	0.4415 (3)	0.25215 (19)	0.8761 (2)	0.0243 (7)	
H7A	0.4148	0.2919	0.9303	0.029*	
H7B	0.3962	0.2743	0.7958	0.029*	
C8	0.3482 (3)	0.1418 (2)	0.9806 (2)	0.0251 (7)	
C9	0.2885 (3)	0.0507 (2)	0.9689 (2)	0.0264 (7)	
H9A	0.1830	0.0522	0.9514	0.032*	
H9B	0.3313	0.0180	1.0425	0.032*	
C10	0.3402 (3)	0.10667 (19)	0.7831 (2)	0.0210 (7)	
C11	0.1909 (3)	0.13109 (19)	0.7013 (2)	0.0227 (7)	
H11A	0.1912	0.1928	0.6780	0.027*	
H11B	0.1202	0.1248	0.7436	0.027*	
C12	0.1448 (3)	0.07408 (18)	0.5916 (2)	0.0212 (7)	
H12A	0.0491	0.0928	0.5400	0.025*	
H12B	0.1375	0.0128	0.6140	0.025*	
C13	0.2545 (3)	0.08156 (19)	0.5260 (2)	0.0213 (7)	
H13A	0.2647	0.1449	0.5116	0.026*	
C14	0.4012 (3)	0.0504 (2)	0.6074 (2)	0.0242 (7)	
H14A	0.3940	−0.0113	0.6284	0.029*	
H14B	0.4731	0.0543	0.5661	0.029*	
C15	0.4503 (3)	0.10522 (19)	0.7182 (2)	0.0221 (7)	
H15A	0.4679	0.1655	0.6975	0.027*	
H15B	0.5421	0.0817	0.7707	0.027*	
C16	0.2087 (3)	0.0375 (2)	0.4081 (2)	0.0217 (7)	
C17	0.2160 (3)	−0.05266 (19)	0.3972 (2)	0.0232 (7)	
H17A	0.2473	−0.0877	0.4655	0.028*	
C18	0.1782 (3)	−0.0918 (2)	0.2878 (2)	0.0260 (7)	
H18A	0.1843	−0.1532	0.2818	0.031*	
C19	0.1316 (3)	−0.0417 (2)	0.1874 (2)	0.0277 (7)	
H19A	0.1060	−0.0684	0.1127	0.033*	
C20	0.1226 (3)	0.0480 (2)	0.1970 (2)	0.0282 (8)	
H20A	0.0910	0.0829	0.1286	0.034*	
C21	0.1598 (3)	0.0866 (2)	0.3064 (2)	0.0248 (7)	
H21A	0.1518	0.1478	0.3120	0.030*	

### Atomic displacement parameters (Å^2^)

**Table:**

	*U*^11^	*U*^22^	*U*^33^	*U*^12^	*U*^13^	*U*^23^
S1	0.0414 (5)	0.0194 (4)	0.0179 (4)	−0.0007 (4)	0.0134 (3)	0.0011 (4)
O1	0.0528 (15)	0.0301 (13)	0.0176 (11)	−0.0009 (11)	0.0177 (10)	−0.0058 (10)
N1	0.0318 (15)	0.0173 (13)	0.0172 (12)	−0.0043 (11)	0.0121 (10)	−0.0026 (11)
C1	0.041 (2)	0.0168 (16)	0.0145 (14)	−0.0017 (14)	0.0096 (14)	−0.0039 (13)
C2	0.049 (2)	0.0215 (18)	0.0238 (16)	−0.0070 (15)	0.0188 (15)	−0.0078 (14)
C3	0.0326 (19)	0.0268 (19)	0.0346 (18)	−0.0060 (14)	0.0125 (15)	−0.0142 (16)
C4	0.042 (2)	0.0256 (18)	0.0227 (16)	0.0024 (15)	0.0071 (15)	−0.0057 (14)
C5	0.041 (2)	0.0177 (17)	0.0195 (16)	−0.0028 (14)	0.0112 (14)	−0.0019 (14)
C6	0.0362 (19)	0.0131 (15)	0.0161 (14)	−0.0020 (13)	0.0111 (13)	−0.0019 (13)
C7	0.042 (2)	0.0149 (16)	0.0182 (15)	0.0026 (14)	0.0122 (13)	−0.0004 (13)
C8	0.0335 (19)	0.0257 (18)	0.0194 (15)	0.0028 (14)	0.0134 (13)	0.0015 (14)
C9	0.0354 (19)	0.0299 (19)	0.0162 (15)	−0.0036 (15)	0.0114 (13)	0.0005 (14)
C10	0.0338 (18)	0.0181 (16)	0.0142 (14)	−0.0021 (13)	0.0122 (13)	−0.0035 (13)
C11	0.0289 (18)	0.0246 (18)	0.0190 (15)	0.0016 (13)	0.0138 (13)	−0.0006 (13)
C12	0.0288 (18)	0.0197 (17)	0.0169 (14)	0.0002 (13)	0.0099 (12)	−0.0003 (13)
C13	0.0315 (18)	0.0196 (16)	0.0155 (14)	−0.0035 (13)	0.0112 (13)	−0.0025 (13)
C14	0.0309 (18)	0.0252 (17)	0.0208 (15)	−0.0009 (14)	0.0144 (13)	−0.0035 (14)
C15	0.0281 (18)	0.0228 (17)	0.0174 (14)	−0.0017 (13)	0.0101 (13)	−0.0017 (13)
C16	0.0262 (17)	0.0228 (16)	0.0192 (15)	−0.0034 (13)	0.0114 (12)	−0.0019 (14)
C17	0.0342 (18)	0.0212 (18)	0.0172 (15)	−0.0030 (14)	0.0125 (13)	0.0020 (14)
C18	0.0331 (19)	0.0217 (17)	0.0261 (16)	−0.0024 (14)	0.0139 (14)	−0.0054 (14)
C19	0.0327 (19)	0.035 (2)	0.0165 (15)	−0.0020 (15)	0.0094 (13)	−0.0069 (15)
C20	0.037 (2)	0.032 (2)	0.0176 (16)	0.0040 (15)	0.0103 (14)	0.0027 (15)
C21	0.0339 (19)	0.0221 (17)	0.0207 (16)	0.0000 (14)	0.0122 (13)	−0.0024 (14)

### Geometric parameters (Å, º)

**Table:**

S1—C9	1.808 (3)	C11—C12	1.532 (4)
S1—C10	1.849 (3)	C11—H11A	0.9900
O1—C8	1.231 (3)	C11—H11B	0.9900
N1—C8	1.360 (3)	C12—C13	1.534 (4)
N1—C7	1.466 (4)	C12—H12A	0.9900
N1—C10	1.476 (3)	C12—H12B	0.9900
C1—C2	1.385 (4)	C13—C16	1.510 (4)
C1—C6	1.394 (4)	C13—C14	1.538 (4)
C1—H1A	0.9500	C13—H13A	1.0000
C2—C3	1.385 (4)	C14—C15	1.524 (4)
C2—H2A	0.9500	C14—H14A	0.9900
C3—C4	1.391 (4)	C14—H14B	0.9900
C3—H3A	0.9500	C15—H15A	0.9900
C4—C5	1.385 (4)	C15—H15B	0.9900
C4—H4A	0.9500	C16—C21	1.390 (4)
C5—C6	1.387 (4)	C16—C17	1.397 (4)
C5—H5A	0.9500	C17—C18	1.391 (4)
C6—C7	1.510 (4)	C17—H17A	0.9500
C7—H7A	0.9900	C18—C19	1.385 (4)
C7—H7B	0.9900	C18—H18A	0.9500
C8—C9	1.510 (4)	C19—C20	1.390 (4)
C9—H9A	0.9900	C19—H19A	0.9500
C9—H9B	0.9900	C20—C21	1.387 (4)
C10—C15	1.527 (4)	C20—H20A	0.9500
C10—C11	1.530 (4)	C21—H21A	0.9500
			
C9—S1—C10	90.75 (13)	C10—C11—H11B	109.2
C8—N1—C7	121.1 (2)	C12—C11—H11B	109.2
C8—N1—C10	117.3 (2)	H11A—C11—H11B	107.9
C7—N1—C10	120.8 (2)	C11—C12—C13	110.0 (2)
C2—C1—C6	120.9 (3)	C11—C12—H12A	109.7
C2—C1—H1A	119.5	C13—C12—H12A	109.7
C6—C1—H1A	119.5	C11—C12—H12B	109.7
C1—C2—C3	120.0 (3)	C13—C12—H12B	109.7
C1—C2—H2A	120.0	H12A—C12—H12B	108.2
C3—C2—H2A	120.0	C16—C13—C12	114.0 (2)
C2—C3—C4	119.6 (3)	C16—C13—C14	113.4 (2)
C2—C3—H3A	120.2	C12—C13—C14	108.4 (2)
C4—C3—H3A	120.2	C16—C13—H13A	106.8
C5—C4—C3	120.0 (3)	C12—C13—H13A	106.8
C5—C4—H4A	120.0	C14—C13—H13A	106.8
C3—C4—H4A	120.0	C15—C14—C13	110.9 (2)
C4—C5—C6	120.9 (3)	C15—C14—H14A	109.5
C4—C5—H5A	119.5	C13—C14—H14A	109.5
C6—C5—H5A	119.5	C15—C14—H14B	109.5
C5—C6—C1	118.5 (3)	C13—C14—H14B	109.5
C5—C6—C7	121.0 (3)	H14A—C14—H14B	108.0
C1—C6—C7	120.5 (3)	C14—C15—C10	112.5 (2)
N1—C7—C6	113.5 (2)	C14—C15—H15A	109.1
N1—C7—H7A	108.9	C10—C15—H15A	109.1
C6—C7—H7A	108.9	C14—C15—H15B	109.1
N1—C7—H7B	108.9	C10—C15—H15B	109.1
C6—C7—H7B	108.9	H15A—C15—H15B	107.8
H7A—C7—H7B	107.7	C21—C16—C17	118.0 (3)
O1—C8—N1	124.7 (3)	C21—C16—C13	120.3 (3)
O1—C8—C9	123.6 (3)	C17—C16—C13	121.7 (2)
N1—C8—C9	111.6 (2)	C18—C17—C16	120.9 (3)
C8—C9—S1	106.9 (2)	C18—C17—H17A	119.6
C8—C9—H9A	110.3	C16—C17—H17A	119.6
S1—C9—H9A	110.3	C19—C18—C17	120.3 (3)
C8—C9—H9B	110.3	C19—C18—H18A	119.9
S1—C9—H9B	110.3	C17—C18—H18A	119.9
H9A—C9—H9B	108.6	C18—C19—C20	119.4 (3)
N1—C10—C15	111.8 (2)	C18—C19—H19A	120.3
N1—C10—C11	110.7 (2)	C20—C19—H19A	120.3
C15—C10—C11	111.3 (2)	C21—C20—C19	120.0 (3)
N1—C10—S1	102.67 (17)	C21—C20—H20A	120.0
C15—C10—S1	110.1 (2)	C19—C20—H20A	120.0
C11—C10—S1	110.0 (2)	C20—C21—C16	121.4 (3)
C10—C11—C12	112.0 (2)	C20—C21—H21A	119.3
C10—C11—H11A	109.2	C16—C21—H21A	119.3
C12—C11—H11A	109.2		
			
C6—C1—C2—C3	1.3 (4)	C9—S1—C10—C15	148.8 (2)
C1—C2—C3—C4	−2.3 (4)	C9—S1—C10—C11	−88.2 (2)
C2—C3—C4—C5	2.0 (4)	N1—C10—C11—C12	177.3 (2)
C3—C4—C5—C6	−0.6 (4)	C15—C10—C11—C12	52.4 (3)
C4—C5—C6—C1	−0.4 (4)	S1—C10—C11—C12	−69.9 (3)
C4—C5—C6—C7	−179.8 (3)	C10—C11—C12—C13	−57.8 (3)
C2—C1—C6—C5	0.1 (4)	C11—C12—C13—C16	−172.1 (2)
C2—C1—C6—C7	179.4 (3)	C11—C12—C13—C14	60.4 (3)
C8—N1—C7—C6	101.0 (3)	C16—C13—C14—C15	172.6 (2)
C10—N1—C7—C6	−89.9 (3)	C12—C13—C14—C15	−59.6 (3)
C5—C6—C7—N1	−47.5 (3)	C13—C14—C15—C10	55.7 (3)
C1—C6—C7—N1	133.2 (3)	N1—C10—C15—C14	−175.6 (2)
C7—N1—C8—O1	−1.7 (5)	C11—C10—C15—C14	−51.3 (3)
C10—N1—C8—O1	−171.2 (3)	S1—C10—C15—C14	71.0 (3)
C7—N1—C8—C9	178.6 (2)	C12—C13—C16—C21	104.1 (3)
C10—N1—C8—C9	9.1 (4)	C14—C13—C16—C21	−131.1 (3)
O1—C8—C9—S1	−164.6 (3)	C12—C13—C16—C17	−77.1 (4)
N1—C8—C9—S1	15.1 (3)	C14—C13—C16—C17	47.8 (4)
C10—S1—C9—C8	−26.3 (2)	C21—C16—C17—C18	1.2 (4)
C8—N1—C10—C15	−145.8 (3)	C13—C16—C17—C18	−177.7 (3)
C7—N1—C10—C15	44.7 (3)	C16—C17—C18—C19	−0.4 (4)
C8—N1—C10—C11	89.6 (3)	C17—C18—C19—C20	−0.2 (4)
C7—N1—C10—C11	−79.9 (3)	C18—C19—C20—C21	−0.2 (4)
C8—N1—C10—S1	−27.8 (3)	C19—C20—C21—C16	1.0 (5)
C7—N1—C10—S1	162.7 (2)	C17—C16—C21—C20	−1.5 (4)
C9—S1—C10—N1	29.6 (2)	C13—C16—C21—C20	177.4 (3)

### Hydrogen-bond geometry (Å, º)

Cg1 is the centroid of the C1–C6 ring.

**Table:**

*D*—H···*A*	*D*—H	H···*A*	*D*···*A*	*D*—H···*A*
C17—H17*A*···*Cg*1^i^	0.95	2.72	3.565 (3)	149

Symmetry code: (i) −*x*+1, *y*−1/2, −*z*+3/2.
